# The number and genetic relatedness of transmitted/founder virus impact clinical outcome in vaginal R5 SHIV_SF162P3N_ infection

**DOI:** 10.1186/1742-4690-11-22

**Published:** 2014-03-11

**Authors:** Lily Tsai, Ivan Tasovski, Ana Rachel Leda, Mario PS Chin, Cecilia Cheng-Mayer

**Affiliations:** 1 Aaron Diamond AIDS Research Center, Aaron Diamond Professor at the Rockefeller University, New York, NY, USA; 2 Department of Microbiology and Immunology, Temple University School of Medicine, Philadelphia, PA, USA; 3 Center for Substance Abuse Research, Temple University School of Medicine, Philadelphia, PA, USA

**Keywords:** Vaginal transmission, Transmitted/founder virus, Quasispecies complexity

## Abstract

**Background:**

Severe genetic bottleneck occurs during HIV-1 sexual transmission whereby most infections are initiated by a single transmitted/founder (T/F) virus. Similar observations had been made in nonhuman primates exposed mucosally to SIV/SHIV. We previously reported variable clinical outcome in rhesus macaques inoculated intravaginally (ivg) with a high dose of R5 SHIV_SF162P3N_. Given the potential contributions of viral diversity to HIV-1 persistence and AIDS pathogenesis and recombination between retroviral genomes increases the genetic diversity, we tested the hypothesis that transmission of multiple variants contributes to heightened levels of virus replication and faster disease progression in the SHIV_SF162P3N_ ivg-infected monkeys.

**Results:**

We found that the differences in viral replication and disease progression between the transiently viremic (TV; n = 2), chronically-infected (CP; n = 8) and rapid progressor (RP; n = 4) ivg-infected macaques cannot be explained by which variant in the inoculum was infecting the animal. Rather, transmission of a single variant was observed in both TV rhesus, with 1–2 T/F viruses found in the CPs and 2–4 in all four RP macaques. Moreover, the genetic relatedness of the T/F viruses in the CP monkeys with multivariant transmission was greater than that seen in the RPs. Biological characterization of a subset of T/F envelopes from chronic and rapid progressors revealed differences in their ability to mediate entry into monocyte-derived macrophages, with enhanced macrophage tropism observed in the former as compared to the latter.

**Conclusion:**

Our study supports the tenet that sequence diversity of the infecting virus contributes to higher steady-state levels of HIV-1 virus replication and faster disease progression and highlights the role of macrophage tropism in HIV-1 transmission and persistence.

## Background

The human immunodeficiency virus type 1 (HIV-1) is composed of swarms of related viruses, forming what are known as viral quasispecies [1-4]. HIV-1 heterogeneity is the result of the high error rate and lack of proof-reading mechanisms of reverse transcriptase [5-7] as well as genetic recombination between the retroviral genomes [8]. Quasispecies diversity after primary infection has been implicated in the pathogenesis of polioviruses [9,10], the West Nile virus [11] and HIV [12-15]. Acute infection with heterogeneous HIV populations has also been suggested to promote viral persistence and rates of disease development [16-20]. It is hypothesized that through recombination and cooperative interactions, the viral quasispecies provide greater probability to evolve and escape the changing host selective pressures during early infection, with consequences for viral pathogenesis and therapy [21]. Indeed, it has recently been reported that recombination occurs frequently and rapidly *in vivo*, replacing the parental T/F viral populations within four months of infection [22].

In this regard, severe genetic bottleneck occurs during HIV-1 transmission. Most heterosexual mucosal infections are initiated with a single transmitted virus [23], with higher multiplicity of infection seen in men who have sex with men (~40%) and intravenous drug users (~60%), consistent with the relative risks of transmission via these routes [24,25]. Similar observations had been made in rhesus macaques (RMs) exposed intrarectally (ir) or intravaginally (ivg) to low-dose SIV and SHIV [26-29], and in depo-provera treated macaques exposed vaginally to a single supra-physiological dose of R5 SHIV [30], highlighting the effectiveness of the transmission bottleneck. Given the potential contributions of viral diversity to HIV-1 persistence and AIDS pathogenesis, the restriction in quasispecies population diversity in the recipient hosts upon HIV-1 transmission, in particular via vaginal exposure, could prove disadvantageous to viral persistence and disease induction in the new host. The initial process of fitness recovery therefore may require early and rapid diversification of the transmitted viruses to combat the evolving host selection pressures. This could be achieved through viral turnover of a highly infectious transmitted virus. Alternatively, since recombination between retroviral genomes is estimated to exceed the rate of mutation [31-34], and extensive recombination among HIV-1 quasispecies has been shown to contribute to viral diversity in infected patients [22,35], it is reasonable to hypothesize that transmission of multiple variants facilitates the generation of genetic variations and increase in viral fitness, leading to heightened levels of virus replication and rapid disease progression. We tested this hypothesis by investigating the population size and characteristics of the T/F viruses in R5 SHIV_SF162P3N_ intravaginally infected macaques with variable clinical outcome.

## Results

### Variable disease outcome in macaques infected intravaginally with SHIV_SF162P3N_

**Figure 1:**
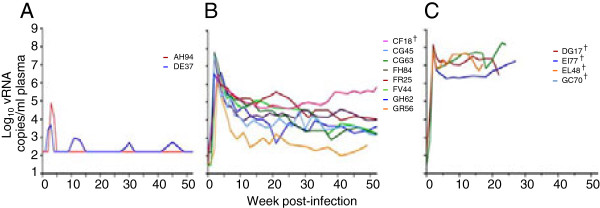
**Plasma viremia over time in R5 SHIV**_**SF162P3N**_**-ivg infected macaques.** Viral load in **(A)** Transiently viremic (TV, n = 2), **(B)** chronic progressor (CP, n = 8) and **(C)** rapid progressor (RP, n = 4) rhesus infected with the same batch of R5 SHIV_SF162P3N_ virus stock is shown. The animals were not synchronized with respect to the menstrual cycle stage prior to ivg challenge. † indicates euthanasia with clinical symptoms of AIDS.

**Figure 2:**
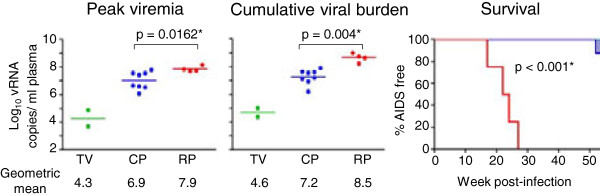
**Comparison of peak, cumulative viral load and survival in R5 SHIV**_**SF162P3N**_**-ivg infected macaques.** Mean peak, cumulative viral burden (computed as an integration of the area under the curve) and survival over a one-year infection period in the transiently viremic (TV; in green), chronically-infected (CP; in blue) and rapid progressor (RP; in red) ivg-infected macaques were compared. An asterisk (*) indicates statistical significance (p < 0.05).

We previously observed variable clinical outcome in rhesus macaques (RMs) infected intravaginally (ivg) with high dose R5 SHIV_SF162P3N_[36]. The animals were not synchronized with respect to the stage of the menstrual cycle before ivg challenge, and were inoculated with different doses (1,000 and 10,000 TCID_50_) and exposure frequencies (once or twice within the same day) using the same batch of virus stock (Table 1). Two ivg-inoculated macaques showed transient viremia (Figure 1A), while establishment of chronic infection in eight (Figure 1B) and rapid disease progression in four was observed (Figure 1C). A dose-dependency in ivg infection outcome was not observed in this small cohort of animals studied. Moreover, a paradoxical inverse association between exposure frequencies and clinical outcome was noted: a rapid progressor (RP) phenotype was absent in the four macaques receiving two high dose virus inoculations four hours apart, with animals either showing transient viremia (AH94, DE37) or slow disease progression (CF18, FH84). This inverse association between exposure frequencies and clinical outcome could not be explained by MHC class I and TRIM5α genetic polymorphisms or selection for particular T/F viruses ([36]; this study), and is seen only with intravaginal and not with intrarectal challenge, raising the possibility of differential anatomical host response to the virus dose or nonviral constituents present in the virus supernatants that could potentially influence viral infectivity and early infection events. Studies in additional animals will be required to address this. As anticipated, peak and cumulative viral load up to the time of euthanasia or over a one-year infection period were significantly higher in the RPs than the chronic progressors (CPs) (p = 0.0162 and p = 0.004 respectively; Figure 2). The four RPs succumbed to AIDS within 30 weeks of infection in the absence of seroconversion, while all eight chronic progressors and one of the transient viremic (TV) animals (AH94) seroconverted at 4–6 week post-infection (wpi). Seven of eight CP and both TV animals remained AIDS-free after one year of infection. The difference in survival between the RPs and CPs is statistically significant (p <0.001; Figure 2).

**Table 1:** **Clinical outcome of macaques infected intravaginally with R5 SHIV**_**SF162P3N**_

**Clinical status**	**Animal**	**Challenge dose; frequency**	**Time to necropsy (weeks)**
Transient Viremic	AH94	10,000; 2X	54
	DE37	10,000; 2X	59
Chronic progressor	CG45	1,000; 1X	59
	CG63	1,000; 1X	59
	FR25	10,000; 1X	129
	FV44	10,000; 1X	53
	GH62	10,000; 1X	53
	GR56	10,000; 1X	53
	CF18	10,000; 2X	52*
	FH84	10,000; 2X	104*
Rapid progressor	DG17	1,000; 1X	22*
		GC70	1,000; 1X	24*
	EI77	10,000; 1X	27*	
	EL48	10,000; 1X	17*	

*AIDS-related euthanasia.

### Faster disease progression correlates with increase number and complexity of transmitted founder viruses

**Figure 3:**
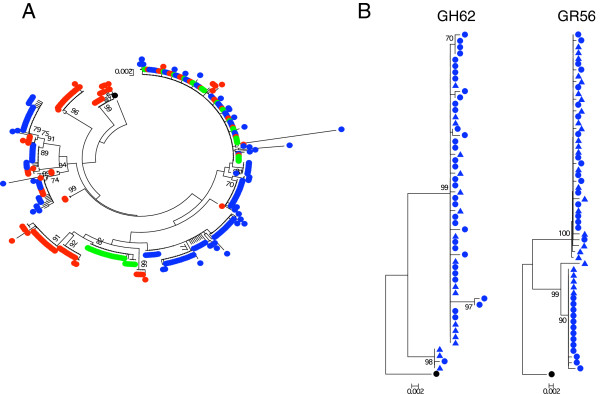
**Phylogenetic tree analyses of T/F *env *from R5 SHIV**_**SF162P3N**_**-ivg infected macaques. (A)** Neighbor-joining tree of *env* V3-V5 sequences from each of the TV (green circle), CP (blue circle) and RP (red circle) macaque is shown. A total of 485 *env* V3-V5 sequences were analyzed (an average of 35 sequences per animal; range, 19 to 59). **(B)** Comparison of *env* V3-V5 sequences of GR56 and GH62 T/F viruses obtained by conventional PCR/cloning (●) and SGA (▲). Sequences are rooted to SF162.

Consistent with our preliminary findings in the CP FH84 and TV DE37 [36], phylogenetic tree analysis of *env* V3-V5 sequences in the first viral RNA positive plasma samples of the ivg-infected animals shows that those from the TV, CP and RP macaques intermingled (Figure 3A), suggesting that the differences in viral replication and disease progression among these three groups of animals cannot be explained by transmission/infection with specific genotypic variants. Because conventional nested PCR and cloning was employed to characterize most *env* sequences, we performed single genome amplification (SGA) and direct sequencing of uncloned *env* amplicons from early plasma of two infected macaques (GR56, GH62) to address the concerns that the results observed may be due to *Taq*-induced PCR errors. Data showed that the *env* sequences obtained with the SGA approach are similar to those obtained by conventional PCR (Figure 3B), consistent with reports that bulk sequencing captures a measure of population diversity similar to that determined by SGA [37].

**Figure 4:**
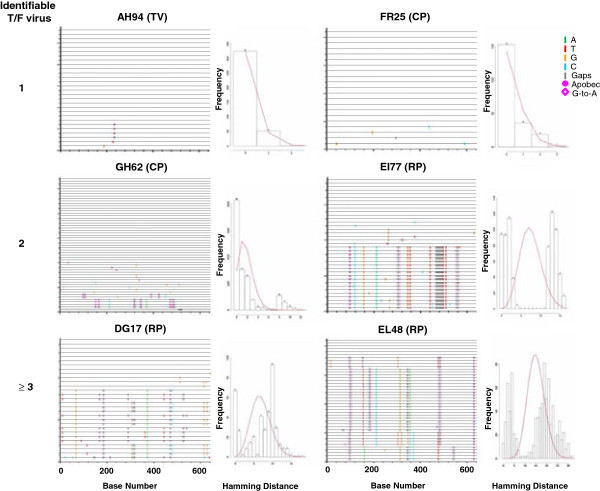
**Highlighter plot and hamming distance analyses of T/F *env *in R5 SHIV**_**SF162P3N **_**ivg-infected macaques.** Data for *env* V3-V5 sequences from representative transiently viremic (TV; AH84), chronically-infected (CP; FR25, GH62) or rapidly-progressing (RP; EI77, DG17, EL48) macaques is shown. Positions of nucleotide base transitions and transversions in the highlighter plots are indicated by short, colored coded bars. Colors are as follows: A: green, T: red, G: yellow. C: blue and gaps: grey. Tics that are bracketed represent G-to-A changes while those in circles represent G-to-A changes in a sequence consistent with an APOBEC signature. The number of identifiable T/F viruses is listed on the left.

**Figure 5:**
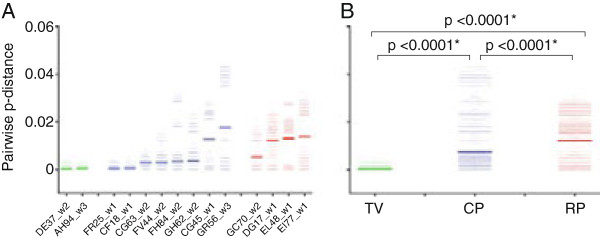
**Pairwise distance analysis of T/F *env *in R5 SHIV**_**SF162P3N **_**ivg-infected macaques.** Data for *env* V3-V5 sequences from all individual macaque **(A)** and among the TV (in green), CP (in blue) and RP (in red) groups **(B)** is shown. The sampling time (wpi) for each animal in the three groups is indicated in **(A)**. The lines in bold in **(A)** and **(B)** represent the mean pairwise distance for each individual animal or group, respectively, with an asterisk (*) in **(B)** indicating statistical significance (p < 0.05).

Primary infection with multiple HIV variants from a single source as determined by heteroduplex mobility tracking of proviral DNA had been suggested to accelerate rates of disease development in human [19], but the degree of population diversity and the number of transmitted variants were not investigated in this study. Accordingly, we determined the number of transmitted/founder viruses in the ivg-infected macaques. Consistent with reports in HIV transmission in human and SIV transmission in macaques [17,23,27,29,38], vaginal transmission of SHIV_SF162P3N_ in RMs is characterized by a genetic bottleneck, with a single or limited number of viral variants transmitted despite the use of high inoculum doses and increased frequency of exposure in several animals. Enumeration of the number of transmitted variants by Highlighter plot analysis shows however that more variants were transmitted in the RP than in the CP or TV macaques (Figure 4). Both TV animals were infected with a single variant, with 1–2 transmitted variants found in CPs and 2–4 variants in the four RP monkeys. The difference in the number of transmitted variants between the latter two groups of animals approached statistical significance (p = 0.0727). Furthermore, pairwise distance analysis shows that T/F variants are more genetically diverse in the RP than in the CP and TV animals (Figure 5). Overall diversity, expressed as the percent of the average pairwise difference in T/F *env* V3-V5 sequences is greater in the RP than the CP and TV. Mean percentage sequence variation among the T/F viruses of TV is 0.050%, and this increases to 0.549% in the CPs and 1.105% in the RPs, with statistically significant differences among the groups that support a positive correlation between the heterogeneity of the infecting virus population and subsequent clinical outcome (p < 0.0001; Figure 5B). Table 2 summarizes the estimated number and genetic diversity of T/F variants in R5 SHIV_SF162P3N_ ivg-infected macaques, showing that virus replication and disease progression in R5 SHIV_SF162P3N_ ivg-infected macaques are associated with the population size and *env* sequence diversity of the transmitter/founder (T/F) viruses.

**Table 2:** **Summary of the estimated number and genetic diversities of T/F variants in SHIV**_**SF162P3N **_**ivg-infected macaques**

**Clinical status**	**Animal**	**Sampling time point (wpi)**	**vRNA (copies/ml plasma) at time of sampling**	**No. of *env *** **sequences analyzed**	**No. of identified T/F viruses**	**Mean *env *** **diversity**
Transient Viremic	DE37	2	2,939	44	1	0.0004
	AH94	3	74,929	28	1	0.0006
Chronic Progressors	FR25	1	2,458	21	1	0.0005
	CF18	1	1,413	19	1	0.0006
	CG63	2	61,996,000*	20	1	0.0030
	FV44	2	39,494	30	2	0.0030
	FH84	2	36,073,000*	49	2	0.0032
	GH62	2	3,902,206*	59^#^	2	0.0034
	CG45	1	7,140	24	2	0.0126
	GR56	3	1,186,674*	58^#^	2	0.0176
Rapid Progressors	GC70	2	67,857,860*	24	2	0.0052
	DG17	1	10,844	29	3	0.0122
	EL48	1	48,548	42	4	0.0130
	EI77	1	17,119	38	2	0.0138

*indicates time of peak viremia; # designates the two animals (GH62, GR56) where both SGA and conventional cloning was performed to generate the *env* sequences: 19 SGA and 40 cloning sequences for GH62 and 27 SGA and 31 cloning sequences for GR56 were analyzed. All the analyses were performed with a 660 bp *env* V3-V5 sequences.

### T/F viruses in chronic progressors are more macrophage-tropic than those in rapid progressors

**Figure 6:**
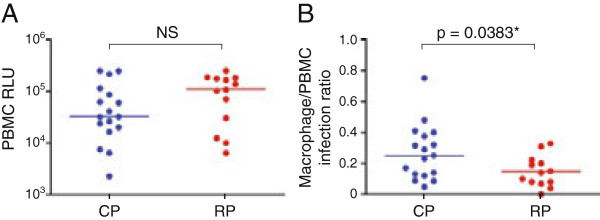
**PBMC (A) and MDM tropism (B) of T/F Envs.** Pseudotype viruses bearing T/F Envs from six CP (in blue) and four RP (in red) macaques are tested for their ability to infect peripheral blood mononuclear cells (PBMCs) and monocyte-derived macrophages (MDMs). To control for differences in viral entry, infectivity in macrophages was normalized to that achieved in PBMCs from the same donor. Data shown are for individual Env clones (2–5) from each animal and is the average of at least two independent experiments. The lines represent the median value for each group and an asterisk (*) indicates statistical significance (p < 0.05). NS, not significant; RLU, relative light units.

HIV-1 mucosal transmission is primarily associated with CD4+ T cell tropism and CCR5 use, with lower levels of replication in monocyte-derived macrophages (MDM) compared to CD4+ T cells for subtype C and B transmitted viruses [24,39]. Because infected macrophages are long-lived [40,41] and resistant to CTL suppression [42,43], and HIV-1 particles within infected macrophages are protected from neutralization antibodies [44-46] and can be transmitted efficiently to T cells [47,48], we compared infection of PBMCs and MDMs mediated by representative Envs from each of the T/F clusters in six of eight CP and all four RP macaques to determine if the latter plays a role in post-acute infection. Results showed no significant difference in the ability of the Envs from the two groups of infected macaques to mediate entry into mitogen-stimulated PBMCs that express high amounts of CD4 and CCR5 (Figure 6A). In contrast, while a wider range of macrophage tropism was seen in the CPs than in the RPs, T/F viruses in RPs overall are less macrophage-tropic than the CPs, with the difference being statistical significance (p = 0.0383; Figure 6B).

## Discussion

By characterizing a cohort of R5 SHIV_SF162P3N_-ivg infected macaques that display distinct clinical outcomes, here we show that a specific viral variant in the challenge stock was not consistently transmitted by intravaginal inoculation and the relative dose of the inoculum did not correlate grossly with the number of variants transmitted. Transmission of multiple and genetically diverse viral variants from the inoculum however is associated with higher peak and chronic viral load as well as accelerated rates of disease development. Moreover, macrophage tropism of the transmitted/founder virus contributes to the establishment of a persistent infection.

A correlation between peak viral load and the number of transmitted viruses was observed for macaques infected intrarectally with SIVsmE660 and SIVmac251 [26], a finding that is recapitulated here in our R5 SHIV vaginal transmission model in which SHIV_SF162P3N_-infected RPs had the highest peak viremia and numbers of T/F viruses. Furthermore, co-infection with divergent HIV-1 subtypes has been associated with more severe disease progression in human [16,18], and heteroduplex tracking assay analysis showed that women who acquired multiple variants from a single source had a significantly higher chronic viral load and lower CD4+ T cell count compared to women who were infected with a single viral genotype [19]. Our study extends these early observations by showing that the number of variants, sequence diversity and macrophage tropism of the T/F populations also contribute to higher steady-state levels of HIV-1 virus replication and faster disease progression. Since viral diversity was examined, in most cases, at the first vRNA positive time point (1–2 weeks post-challenge), the finding of higher levels of acute viremia (2–3 wpi) in monkeys with genetically diverse viruses is most likely due to transmission of multiple envelope genotypes and not the result of viral turnover of highly infectious transmitted viruses. Indeed, we did not find statistically significant differences in the ability of T/F Envs from the CP and RP macaques to mediate entry into mitogen-stimulated PBMCs that would be suggestive of differences in the infectiousness of their T/F viruses. Collectively our data establishes a role of T/F quasispecies diversity in HIV-1 pathogenesis.

The number of T/F variants in our study is likely to be underestimated, since only the gp120 V3-V5 sequences were analyzed. Nonetheless, an association between transmission of diverse population of SHIV variants, higher viral burden and rapid disease progression was seen, supporting a scenario whereby viral quasispecies that are more fit and capable of escaping early host selective pressures are generated through recombination and cooperative interactions between the transmitted variants. Multiple variant transmission represents a significant fraction of transmission events: 24% and 22% for subtype B and C sexually infected men and women, respectively [17,23]. Factors that influence transmission of diverse viruses however are unclear. Biological factors such as gender, viral subtypes, routes of transmission and the presence of STDs can affect multiple variant transmission frequencies. The impact of these factors however is controlled in our study where female macaques were infected via the same route and with the same virus stock in the absence of STDs. Multivariant transmission susceptibility could also be influenced by age, vaginal flora and timing of the menstrual cycle before challenge [49], parameters that were not controlled for in our small cohort study. Moreover, polymorphisms in alpha-interferon (IFN-α) induced restriction factors such as TRIM5-α, APOBEC3G, tetherin and MX2 that affect their expression levels and/or functions may also play a role [50-52]. Indeed, a recent study showed that compared to chronic viruses, subtype B but not subtype C T/F viruses in human are more resistant to IFN-α [53]. However, we did not find any differences in the IFN-α sensitivity of HIV-1 NL4-3 reporter genome pseudotyped with T/F Envs from the RP and CP rhesus (unpublished observations). Studies using full-length infectious T/F molecular clones and in a larger cohort of ivg-infected macaques therefore will be needed to determine the effect of varying biologic and innate host factors in multivariant transmission frequency.

In agreement with a recent report that all T/F viruses replicated in MDM to various levels [54], our study shows variability in the ability of T/F Envs to function with primary macrophages, with those derived from CP macaques mediating more efficient entry than the ones from RP rhesus. These immune cells play a duplicitous role during early HIV-1 infection. Macrophages in the vaginal mucosa have been shown to be productively infected [55], secreting cytokines to recruit CD4+ T cells at the sites of viral entry to fuel the infection [56]. Moreover, infected macrophages possess potent immune evasion mechanisms, are long-lived viral reservoirs and are particularly efficient at transmitting the virus to new CD4+ T cells [57]. At the same time, as antigen presenting cells, macrophages can take up and process virus for priming of CD4+ and CD8+ T cells to initiate and orchestrate antiviral humoral and cellular immune response. Thus, it is conceivable that the inability of the RP macaques to control virus infection is due in part to inefficient macrophage infection of the T/F viruses which hampered the development of effective adaptive immune responses. Conversely, efficient macrophage infection by T/F viruses in the CP rhesus promotes not only viral transmission and spread, but T and B cell responses to reduce acute viremia, leading to the onset of a persistent chronic infection.

## Conclusions

Our study established the view that the population size and genetic complexity of the transmitted virus population impact the subsequent course of R5 SHIV vaginal infection and highlights the role of acute quasispecies diversity and macrophage tropism in

HIV-1 associated pathogenesis. We posit that increased complexity of the T/F populations coupled with inefficient macrophage infection hampers the initiation and orchestration of adaptive immune responses and contributed to the inability of the R5 SHIV_SF162P3N_ ivg-infected RP macaques to control viral replication. It will be of interest to characterize and compare the occurrence, frequency and kinetics of retroviral recombination in CP and RP macaques that are infected with multiple variants to assess the effect of T/F diversity on viral evolution, fitness and host immune response.

## Methods

### Ethical statement

This work used blood from SHIV infected macaques housed at the Tulane National Primate Research Center (TNPRC) in accordance with the animal Welfare Act and Guide for the Care and Use of Laboratory Animals. TNPRC is accredited by the Association and Assessment and Accreditation of Laboratory Animal Care (AAALAC #00594). The OLAW animal welfare assurance number for TNPRC is A4499-01 and the USDA registration number is 72-R-002. All procedures were performed on anesthetized animals and post-operative analgesics were administered as needed in accordance with the Tulane IACUC approval.

### Cells

293 T cells were maintained in DMEM supplemented with 10% fetal bovine serum (FCS), 100 U/ml penicillin, 100 μg/ml streptomycin and 2 mM L-glutamine (complete medium). Human peripheral mononuclear cells (PBMCs) were prepared by Ficoll gradient centrifugation, stimulated with phytohemagglutinin (PHA, 3 μg/ml; Sigma, St. Louis, MO) in RPMI medium containing 10% FCS, penicillin, streptomycin, L-glutamine and 20 U/ml interleukin-2 (Novartis, Emeryville, CA). Monocytes were enriched by centrifugation of PBMCs through a 40% percoll cushion followed by plastic adherence, and cultured in RPMI 1640 medium supplemented with 10% FCS, 5% human AB serum and 25 ng/ml GM-CSF (Invitrogen, Carlsbad, CA) for 5–7 days to allow for differentiation into macrophages.

### Plasmid constructs and pseudotyped virus production

For expression of envelope glycoproteins (Env), viral RNA was prepared from 0.5 – 1 ml plasma using a commercially available RNA extraction kit (Qiagen, Chatsworth, CA) followed by reverse-transcription (RT) with Superscript III RT (Invitrogen) and random hexamer primers (Amersham Pharmacia, Piscataway, NJ). Full-length gp160 coding sequences were amplified from cDNA by single genome amplification (SGA) or by conventional PCR. For SGA, cDNA was titrated by endpoint dilution and a single copy obtained in a two-step nested PCR procedure using Platinum *Taq* High Fidelity polymerase (Invitrogen) and the primers SH50 (5′-TAGAGCCCTGGAAGCATCCAGGAAGTCAGCCTA -3′) and SH51 (5′ –TCCAGTCCCCCCTTTTCTTTTATAAAA -3′), and SH43 (5′-AAGACAGAATTCATGAGAGTGAAGGGGATCAGGAAG -3′) and SH44 (5′-AGAGAGGGATCCTTATAGCAAAGCCCTTTCAAAGCCCT -3′) for the first and second rounds of PCR, respectively. The same primers were used for conventional nested PCR. Amplicons were subcloned into the pCAGGS vector and sequenced, and *trans*-complementation assay was then used to generate luciferase reporter viruses capable of only a single round of replication. Briefly, Env expression plasmid and the NL4.3LucE-R + vector were cotransfected with polyethylenimine (PEI, Polyscience, Warrington, PA) into 2.5 × 10^6^ 293 T cells plated in a 100-mm plate. Cell culture supernatants were harvested 72 hours later, filtered through 0.45-μm filters, and stored at −70°C in 1-ml aliquots. Pseudotyped viruses were quantified for p24 Gag content (Beckman Coulter, Fullerton, CA).

### Virus infectivity

For assessment of entry efficiency into primary cells, 10^5^ and 10^6^ human PBMCs and macrophages respectively were infected in duplicate with 5 ng p24 Gag equivalent of the indicated pseudotype viruses in each well of a 96-well plate. Infected cells were cultured for 72 h at 37°C, at which time the cells were harvested, lysed and processed for luciferase activity according to the manufacturer’s instructions (Luciferase Assay System; Promega, Madison, WI). Entry, as quantified by luciferase activity, was measured with an MLX microtiter plate luminometer (Dynex Technologies, Inc., Chantilly, VA). To control for differences in Env entry efficiencies, infectivity in macrophages was expressed as a ratio of the infectivity for these cells compared to the infectivity in PBMCs from the same donor.

### Phylogenetic and Highlighter Plot analysis of *env* viral sequences

DNA sequences encompassing *env* V3 to V5 region of gp120 (660 bp) were aligned by Clustal W [58], using SF162 strain as reference. A codon-based alignment was also performed in order to remove sequencing errors, gaps and homopolymeric regions. Neighbor-joining phylogenetic trees were generated by MEGA 5.2.2 [59], using the Jukes Cantor model of evolution [60], with a gamma distribution of site-to-site rate variation as estimated by the FindModel tool from the Los Alamos National Laboratory (LANL) HIV Database (http://hiv.lanl.gov). Gaps but not hypermutated sequences were excluded from the analyses. Viral sequences were visually assessed using the Highlighter tool at the LANL HIV Database.

### Hamming and pairwise distance analyses

DNA sequences encompassing the V3 to V5 regions of *env* were first aligned by ClustalW [58]. Consensus sequences were then generated with the Consensus Maker tool provided by the Los Alamos National Laboratory (LANL) HIV Database (http://hiv.lanl.gov). Best fitting Poisson distributions and Hamming distance frequency distributions were then computed utilizing the Poisson-Fitter tool found at http://hiv.lanl.gov[61], whereby no APOBEC correction was applied and mutation rates were adjusted such that the time estimates since the most recent common ancestor best matched the time of sequencing for each animal. Lastly, pairwise distances were calculated from ClustalW-aligned sequences using MEGA 5.2.2 [59].

### Statistical analysis

All statistical analyses were performed using GraphPad Prism (version 6.0; GraphPad Software, San Diego, CA). Differences in time to AIDS onset between groups were assessed using the log-rank test, while differences among groups in peak viremia, cumulative viral load, pairwise distance and cell tropism were examined using the Mann–Whitney two-tailed t tests. A P value of <0.05 was considered to be statistically significant.

## Competing interests

The authors declare no competing interests.

## Authors’ contributions

LT performed the experiments and data analysis. IT and ARL contributed to the analysis and interpretation of the study. CCM and MPSC designed the study, interpreted the data and wrote the manuscript with help and approval from all authors.
